# How sensory-motor systems impact the neural organization for language: direct contrasts between spoken and signed language

**DOI:** 10.3389/fpsyg.2014.00484

**Published:** 2014-05-27

**Authors:** Karen Emmorey, Stephen McCullough, Sonya Mehta, Thomas J. Grabowski

**Affiliations:** ^1^Laboratory for Language and Cognitive Neuroscience, School of Speech, Language, and Hearing Sciences, San Diego State UniversitySan Diego, CA, USA; ^2^Department of Psychology, University of WashingtonSeattle, WA, USA; ^3^Department of Radiology, University of WashingtonSeattle, WA, USA

**Keywords:** American Sign Language, audio-visual English, bimodal bilinguals, PET, fMRI

## Abstract

To investigate the impact of sensory-motor systems on the neural organization for language, we conducted an H_2_^15^O-PET study of sign and spoken word production (picture-naming) and an fMRI study of sign and audio-visual spoken language comprehension (detection of a semantically anomalous sentence) with hearing bilinguals who are native users of American Sign Language (ASL) and English. Directly contrasting speech and sign production revealed greater activation in bilateral parietal cortex for signing, while speaking resulted in greater activation in bilateral superior temporal cortex (STC) and right frontal cortex, likely reflecting auditory feedback control. Surprisingly, the language production contrast revealed a relative increase in activation in bilateral occipital cortex for speaking. We speculate that greater activation in visual cortex for speaking may actually reflect cortical attenuation when signing, which functions to distinguish self-produced from externally generated visual input. Directly contrasting speech and sign comprehension revealed greater activation in bilateral STC for speech and greater activation in bilateral occipital-temporal cortex for sign. Sign comprehension, like sign production, engaged bilateral parietal cortex to a greater extent than spoken language. We hypothesize that posterior parietal activation in part reflects processing related to spatial classifier constructions in ASL and that anterior parietal activation may reflect covert imitation that functions as a predictive model during sign comprehension. The conjunction analysis for comprehension revealed that both speech and sign bilaterally engaged the inferior frontal gyrus (with more extensive activation on the left) and the superior temporal sulcus, suggesting an invariant bilateral perisylvian language system. We conclude that surface level differences between sign and spoken languages should not be dismissed and are critical for understanding the neurobiology of language.

## Introduction

Evidence from lesion-based, neuroimaging, and neurophysiological studies has revealed that the same left perisylvian regions are recruited during the production and comprehension of both spoken and signed languages (for reviews see Emmorey, 2002; MacSweeney et al., 2008a; Corina et al., 2013). Nonetheless, the neural substrates for speech and sign are not identical. In the experiments presented here, we endeavor to identify the specific sensory- and motor-related systems that are differentially recruited for spoken and signed languages within the same individual: hearing bilinguals who are native users of American Sign Language (ASL) and English. We first examine language production using positron emission tomography (PET) and report the first study (to our knowledge) to contrast sign and word production within participant and without reference to a common motoric baseline that would remove the modality effects of interest. We next review previous production studies that identified the neural overlap between signing and speaking in order to provide a complete picture of language produced by hand and by mouth. We then turn to language comprehension and report the results of an fMRI study that directly contrasts sentence comprehension in ASL and English in hearing bilinguals. Finally, we present data from this study that reveals for the first time the neural conjunction for visual sign comprehension and audiovisual speech comprehension.

The goal of these direct contrasts for both language production and comprehension is to target the neural substrates that are specific to visual-manual and auditory-vocal languages. The goal of the conjunction analyses is to identify neural substrates that are common to both language types. By establishing both the differences and similarities between the neural substrates that support spoken and signed language processing, we can characterize the neurobiological impact of using the hands or the vocal tract as the primary linguistic articulators and of the perceptual reliance on vision or audition for language comprehension.

## Experiment 1: contrasting the neural substrate for sign vs. word production

To date, no neuroimaging study has directly contrasted signing and speaking without subtracting activation from a common motor baseline. For example, Emmorey et al. (2007) conducted a between-group comparison of deaf signers and hearing speakers in which participants overtly named pictures in contrast to a baseline task that required a manual or a vocal response. The goal of that group comparison was to investigate similarities and differences between sign and word production at higher levels of lexical processing, using the baseline task in part to eliminate surface-level differences between sign and speech articulation. In fact, Emmorey et al. (2007) reported no neural regions that exhibited greater activity for speech compared to sign when controlling for low-level motoric and sensory differences through the use of baseline tasks.

Braun et al. (2001) contrasted signed and spoken narrative production (spontaneous autobiographical narratives) by hearing ASL-English bilinguals. Like the Emmorey et al. (2007) study, the contrast between the production of English and ASL was conducted with respect to perceptual-motoric baseline tasks for speech (oral movements with vocalizations) and for sign (hand and limb movements). Signing and speaking were not directly contrasted with one another, although the interaction analyses suggested activations that Braun et al. (2001) attributed to modality-dependent features related to articulation. Specifically, for English increased neural activity was observed in prefrontal and subcortical regions, and Braun et al. (2001) hypothesized that greater activity in these regions reflected the more rapid, sequential oral articulations required for speech. ASL production was associated with greater activity in the superior parietal and paracentral lobules, which Braun et al. (2001) attributed to the execution of complex handshapes and movements to various locations on the body. However, Braun et al. (2001) acknowledged that some of these differences might also reflect modality-specific differences at higher levels of processing, such as the syntactic and semantic use of signing space.

By directly contrasting speaking and signing, we can identify what perceptual and articulatory differences are found when the sensory-motor activation related to vocal and manual baselines is not “subtracted out” of the analysis; that is, a direct contrast provides a better assessment of the neural differences that are specifically related to the sensory-motoric properties of sign vs. word production. Further, we can determine whether the sensory-motoric differences identified by Braun et al. (2001) during narrative production also occur during the production of single lexical items.

### Materials and methods

#### Participants

Fourteen ASL-English bilinguals participated in an H_2_^15^O-PET study (9 women; mean age = 27 years). All participants were exposed to ASL from birth from their deaf signing families, reported normal hearing, were right-handed, and had 12 or more years of formal education.

#### Materials and procedure

Participants overtly named pictures (line drawings of objects from Bates et al., 2003) in either ASL or in English. For each language, participants named 80 pictures in four blocks of 20 pictures each; half had high and half had low frequency names^1^. The order of the ASL and English naming conditions was counterbalanced across participants, and each picture was only presented once during the experiment (i.e., half of the participants named a given picture in English and half in ASL). Pictures were presented to participants using I-glasses SVGA Pro goggles (I-O Display Systems; Sacramento, CA). For each naming block, the picture stimuli were presented from 5 s after the injection (approximately 7–10 s before the bolus arrived in the brain) until 35 s after the intravenous bolus injection of 15 mCi of [^15^O]water, and each picture was presented for 1 s followed by a 1 s inter-stimulus-interval.

#### Image acquisition

All participants underwent MR scanning in a 3.0T TIM Trio Siemens scanner to obtain a 3D T1-weighted structural scan with isotropic 1 mm resolution using the following protocol: MP-RAGE, TR 2530, TE 3.09, TI 800, FOV 25.6 cm, matrix 256 × 256 × 208. PET data were acquired with a Siemens/CTI HR+ PET system using the following protocol: 3D, 63 image planes, 15 cm axial FOV, 4.5 mm transaxial and 4.2 mm axial FWHM resolution.

Images of rCBF were computed using the [^15^O]water autoradiographic method (Herscovitch et al., 1983; Hichwa et al., 1995) as follows. Dynamic scans were initiated with each injection and continued for 100 s, during which 20 5-s frames were acquired. To determine the time course of bolus transit from the cerebral arteries, time-activity curves were generated for regions of interest placed over major vessels at the base of the brain. The eight frames representing the first 40 s immediately after transit of the bolus from the arterial pool were summed to make an integrated 40-s count image. These summed images were reconstructed into 2 mm pixels in a 128 × 128 matrix.

#### Spatial normalization

PET data were spatially normalized to a Talairach-compatible atlas through a series of coregistration steps (see Damasio et al., 1994; Grabowski et al., 1995, for details). Prior to registration, the MR data were manually traced to remove extracerebral voxels. Talairach space was constructed directly for each participant via user-identification of the anterior and posterior commissures and the midsagittal plane on the 3D MRI data set in Brainvox. An automated planar search routine defined the bounding box and piecewise linear transformation was used (Frank et al., 1997), as defined in the Talairach atlas. After Talairach transformation, the MR data sets were warped (AIR 5th order non-linear algorithm) to an atlas space constructed by averaging 50 normal Talairach-transformed brains, rewarping each brain to the average, and finally averaging them again, analogous to the procedure described in Woods et al. (1999). Additionally, the MR images were segmented using a validated tissue segmentation algorithm (Grabowski et al., 2000), and the gray matter partition images were smoothed with a 10 mm FWHM Gaussian kernel. These smoothed gray matter images served as the target for registering participants' PET data to their MR images.

For each participant, PET data from each injection were coregistered to each other using Automated Image Registration (AIR 5.25, Roger Woods, UCLA). The coregistered PET data were averaged, and the mean PET image was then registered to the smoothed gray matter partition using FSL (Jenkinson and Smith, 2001; Jenkinson et al., 2002). The deformation fields computed for the MR images were then applied to the PET data to bring them into register with the Talairach-compatible atlas. After spatial normalization, the PET data were smoothed with a 16.1 × 16.1 × 15.0 mm FWHM Gaussian kernel using complex multiplication in the frequency domain to produce a final isotropic voxel resolution of 18 mm. PET data from each injection were normalized to a global mean of 1000 counts per voxel.

#### Regression analysis

PET data were analyzed with a pixelwise general linear model (Friston et al., 1995). Regression analysis was performed using tal_regress, a customized software module based on Gentleman's least squares routines (Miller, 1991) and cross-validated against SAS (Grabowski et al., 1996). The regression model included covariables for task condition (language modality, frequency, and length manipulations) and subject effects. The contrast between signing and speaking was computed using the appropriate linear combination of task conditions. Results were thresholded for a two tailed *t*-test (familywise error rate *p* < 0.05) using random field theory (RFT) to correct for multiple spatial comparisons across the whole brain (Worsley et al., 1992; Worsley, 1994).

### Results

**Figure 1:**
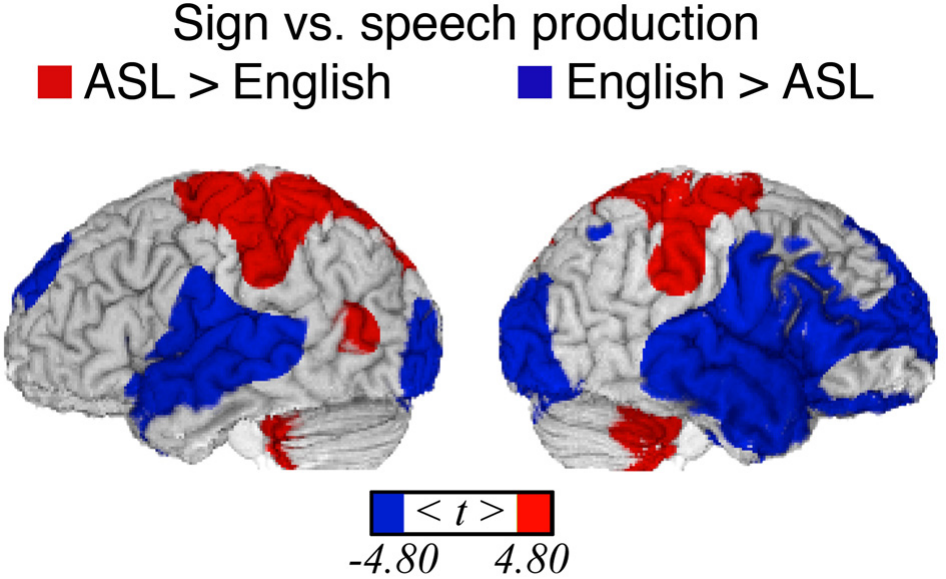
**Significant differences in language production-related activity depending on modality (*p* < 0.05, corrected using RFT) overlaid onto an individual brain**. Surface differences are observed in both primary sensory/motor areas and higher order association cortex. Regions more active for signing (indicated in red) include bilateral sensory-motor areas associated with control of the upper limbs, superior parietal lobule, left middle temporal gyrus (in the vicinity of area MT), and bilateral anterior/inferior cerebellum. Regions more active for speaking (blue) include bilateral sensory-motor areas associated with control of face/mouth, superior temporal, superior frontal, extrastriate visual cortices, and right middle frontal and inferior temporal cortex.

Table 1 provides the local maxima for the direct contrast between sign production and word production, and these results are illustrated in Figure 1. As expected from previous studies, sign production was associated with greater activation in parietal cortices compared to speaking, while speaking resulted in greater activation in bilateral superior temporal cortices, which is most likely due to the auditory feedback that occurs during speaking. In addition, differences within sensory-motor cortices were observed reflecting articulatory differences between signing and speaking. For signing, there was greater activation bilaterally in the cerebellum and in superior regions of the pre- and post-central gyri associated with motor and somatosensory responses for the upper extremities of both limbs. For speaking, there was increased activation in more inferior sensory-motor regions associated with control of the face and mouth. Spoken word production also resulted in increased activation in bilateral middle and superior frontal cortices, compared to sign production.

**Table 1:** **Summary of PET activation results for the comparison between signing and speaking**.

**Region**	**Side**	***X***	***Y***	***Z***	***T***
**SIGNING > SPEAKING**					
**Frontal cortex**					
Pre-central gyrus (BA 6)	R	+27	−17	+59	9.87
**Temporal cortex**					
Mid. temporal gyrus (BA 21)	L	−43	−62	+11	11.99
**Parietal cortex**					
Inferior parietal cortex (BA 40)	R	+56	−29	+38	10.24
Superior parietal lobule (BA 1, 2, 3, 4, 7)	L	−35	−31	+50	22.25
	R	+33	−35	+51	11.03
**Occipital cortex**					
Cuneus (BA 19)	L	−10	−81	+36	5.76
**Subcortical regions**					
Thalamus	L	−9	−19	+2	5.55
**Cerebellum**					
	L	−33	−39	−28	9.67
	R	+18	−45	−19	11.70
**SPEAKING > SIGNING**					
**Frontal cortex**					
Pre-central gyrus (BA 6)	L	−56	−6	+42	−6.16
	R	+58	−2	+40	−8.32
Inferior frontal gyrus (BA 47)	R	+29	+21	−17	−7.51
Middle frontal gyrus (BA 8)	R	+52	+16	+38	−6.16
Middle frontal gyrus (BA 9)	L	−54	+18	+30	−5.12
Inf./Mid. frontal gyrus (BA 46)	L	−52	+36	+8	−4.82
	R	+55	+25	+26	−8.24
Medial frontal gyrus (BA 10)	R	+16	+54	+14	−8.87
Medial frontal gyrus (BA 11)	L	−8	+49	−14	−7.02
Superior frontal gyrus (BA 9)	R	+5	+47	+32	−10.18
Superior frontal gyrus (BA 11)	R	+16	+45	−14	−6.41
**Temporal cortex**					
Superior temporal gyrus	L	−58	−16	+5	−18.89
	R	+63	−10	+7	−18.10
Inferior parietal lobule (BA 40)	R	+47	−60	+48	−5.33
**Occipital cortex**					
Mid. occipital gyrus (BA 18)	R	+32	−93	+15	−10.39
Inf. occipital gyrus (BA 18)	L	−29	−96	−6	−10.57
Lingual gyrus (BA 18)	R	+24	−98	−6	−9.82

*Results are from the whole brain analysis [critical t_(91)_ = ±4.80]*.

Somewhat surprisingly, more extensive activation in bilateral occipital cortex was observed for speaking in contrast to signing. To confirm this unexpected result, we conducted a conjunction analysis using the data from Emmorey et al. (2005) in which a different group of hearing bilinguals named pictures in either ASL or English. In that study, bilinguals viewed line drawings depicting a spatial relation between two objects and produced either an ASL locative classifier construction or an English preposition that described the spatial relation, and the comparison task was to name the figure object (colored red) in either ASL or in English. No motoric baseline was included in this study, and Emmorey et al. (2005) did not report a direct contrast between sign and speech because their focus was the neural correlates of spatial language in ASL compared to English. To compute the contrast between signing and speaking, PET data from the object-naming condition in the Emmorey et al. (2005) study were processed in an essentially identical manner as the current data. Results were thresholded for a two tailed *t*-test (familywise error rate *p* < 0.05, corrected using RFT; Worsley et al., 1992; Worsley, 1994). We used the Minimum Statistic compared to the Conjunction Null method, as described in Nichols et al. (2005) because this type of conjunction analysis is by nature conservative, requiring identified regions to be independently significant in both groups of subjects. This conjunction analysis replicated and confirmed the surprising finding that when directly contrasted, greater activation in bilateral occipital cortex was observed for speaking than for signing (see Supplementary Table).

### Discussion

Differences between the linguistic articulators for speaking and signing were reflected in greater activation along inferior regions of the sensory-motor strip associated with the oral articulators for speech and increased activation in superior regions associated with the arms for sign production. We did not see evidence for greater engagement of the prefrontal corticostriatal-thalamocortical circuit for speech that Braun et al. (2001) hypothesized to be preferentially recruited to control the timing and sequencing of phonetic units when speaking. However, the timing demands for speaking are likely to be greater for connected narratives than for the production of isolated individual words.

Spoken word production results in auditory feedback, which is reflected in more activation within bilateral superior temporal cortex (STC) for speaking. In addition, spoken word production recruited right frontal cortices to a greater extent than sign production (see Figure 1; Table 1). Listening to speech, including self-produced speech, activates right inferior frontal cortex (see Figure 3 below and Tourville et al., 2008), whereas self-produced signing does not result in a visual signal that is parallel to perceiving sign language produced by another person (Emmorey et al., 2009a,b), and self-produced signing does not strongly activate right frontal cortices (e.g., Emmorey et al., 2003; Hu et al., 2011). The activation peak in the right middle frontal gyrus (+52, +16, +38) for speaking (>signing) is near the coordinates for the right lateralized feedback control component for speech production proposed by the DIVA model (Tourville and Guenther, 2011). According to this model, right ventral premotor and right inferior frontal cortex (pars triangularis) receive auditory feedback signals from left and right posterior superior temporal gyri. These right frontal regions mediate between auditory and motor cortices during self-monitoring of speech production. It is unlikely that self-monitoring of sign production relies on this feedback circuit; rather, sign monitoring appears to be more dependent on somatosensory than visual feedback (Emmorey et al., 2009a,b), which likely relies on a fronto-parietal-cerebellar circuit.

The direct contrast between speaking and signing revealed a surprising relative increase in activation within bilateral occipital cortex for speaking. We speculate that greater activation in visual cortex for speaking in contrast to signing may reflect the suppression of activation in these areas when signing. That is, the neural response to self-produced signing within visual cortex may be suppressed, just as the neural response in auditory cortex is suppressed during self-produced speech (e.g., Numminen et al., 1999; Houde et al., 2002). Note that Braun et al. (2001) required participants to close their eyes when speaking and signing, and thus this study would be unable to detect modulations in occipital cortex arising from visual input during language production.

Neural responses to visual input may be generally attenuated during signing in order to help distinguish self-generated motion toward the body from “externally generated” movements of hands or arms toward the body. A signer (or speaker) may be more likely to flinch when another person's hand moves rapidly toward the face or body than when such hand movement is self-produced. Similarly, Hesse et al. (2010) reported cortical attenuation of somatosensory activation elicited by self-produced tactile stimulation and argued that motor commands generate sensory expectations that are compared with the actual sensory feedback to allow for the distinction between internally and externally generated actions. It is possible that posterior parietal cortex and/or left MT (regions that are more active during signing than speaking) may actively inhibit the neural response in occipital cortex to self-generated hand and arm movements during signing. Such modulation could reduce visual attention to self-generated hand movements during signing, and such modulation of occipital cortex would not occur during speaking. However, further investigation is needed to support this speculative hypothesis.

Consistent with several other studies (Braun et al., 2001; Corina and Knapp, 2006; Emmorey et al., 2007), sign production resulted in greater activation in parietal cortex, with more extensive activation in the left hemisphere. The probable source of activation in anterior parietal cortex (including the post-central gyrus) is the somatosensory and proprioceptive feedback received during sign production. Posterior parietal cortex is engaged during the voluntary production of motor movements of the hand and arm, including reaching, grasping, and tool-use (see Creem-Regehr, 2009, for review). Phonological encoding for sign language requires the selection and assembly of one to two hand configurations, locations on the face or body, and movement trajectories. Although inferior parietal cortex is involved in sensory-motor integration during speech production (e.g., Hickok et al., 2009), inferior parietal cortex may play a greater role in sign than speech production. Furthermore, the direct contrast reported here indicates that right inferior parietal cortex is relatively more engaged in sign production (see Figure 1).

Activation in the anterior cerebellum was also greater for signing than speaking, and this region is thought to be involved in sensorimotor processing and prediction of hand and arm actions (e.g., Lorey et al., 2010). Greater cerebellar activity for signing likely reflects the greater demands of on-line motor control for the fingers, hands, and arms. This result is also consistent with recent evidence from diffusion tensor imaging indicating higher fractional anisotropy in the cerebellum for deaf signers relative to hearing non-signers (Tungaraza et al., 2011).

As expected based on previous (non-direct) comparisons between speaking and signing, there was no significant difference between sign and word production in the left inferior frontal gyrus [Brodmann area (BA) 44/45]. There was also no significant difference between language modalities within left posterior temporal cortex, with the exception that sign production engaged left MT to a greater extent than speaking (see Figure 1). Activation in left MT might reflect linguistic processing of hand movements seen in peripheral vision during sign production and/or involvement in phonological encoding of movement parameters for signs. Several studies have found a strong left hemisphere asymmetry for motion processing for signers (both deaf and hearing) compared to non-signers (e.g., Bavelier et al., 2001; Bosworth and Dobkins, 2001).

In sum, when vocal and manual baselines are not included in the direct contrast between speaking and signing, clear modality-related differences in cortical activation emerge. Auditory feedback during speech production engaged STC bilaterally, as well as right inferior frontal cortex. Sign production engaged parietal and cerebellar cortices to a greater extent than speaking, reflecting neural control required to articulate target hand configurations and produce directed movements of the hand and arm toward the body and in space. In addition, the direct contrast between speaking and signing revealed a surprising relative increase in activation within bilateral occipital cortex for speaking, which was confirmed through a conjunction analysis using results from a separate group of ASL-English bilinguals (see Supplementary Table). We speculate that this finding actually reflects the suppression of activation in visual areas when signing, just as neural responses in auditory cortex are suppressed during self-produced speech. Finally, it is worth noting that for spoken language (“unimodal”) bilinguals, the production of their two languages relies on essentially the same neural substrate with few differences, particularly for early simultaneous bilinguals (e.g., Simmons et al., 2011; Parker Jones et al., 2012). The direct contrast between signing and speaking shown in Figure 1 illustrates the rather dramatic difference in neural resources required for the production of a bimodal bilingual's two languages (see Emmorey and McCullough, 2009, for further discussion of the neural consequences of bimodal bilingualism). We now turn to the similarities between language produced by mouth and by hand.

### Common neural substrates for sign and word production

The design of our PET studies with hearing ASL-English bilinguals did not permit a conjunction analysis for speech and sign production because no sensory-motoric or fixation baselines were included (conjunction analyses require a reference baseline). The original questions addressed by our studies required only within condition contrasts between lexical types (e.g., high vs. low frequency items or prepositions vs. nouns), and thus we opted not to include additional injections for a baseline condition. However, other studies have specifically identified the neural overlap for signing and speaking using baseline measures, and we briefly summarize those results.

Braun et al. (2001) asked bimodal bilinguals to produce autobiographical narratives in either English or ASL and to perform non-meaningful complex and simple oral-facial or manual-brachial movements as baseline controls while undergoing PET imaging. Conjunction analyses revealed that discourse production for both languages relied on classical left perisylvian language regions: inferior frontal cortex and posterior STC, extending into middle temporal gyrus. Shared activation for sign and speech production also extended beyond these classical language regions, including left anterior insula, right posterior superior temporal gyrus (STG) extending into the angular gyrus, and bilateral basal temporal cortex (fusiform and lingual gyri).

Braun et al. (2001) suggest that left anterior brain regions [the frontal operculum, insula, lateral premotor cortex, and supplementary motor area (primarily pre-SMA)] are involved the phonological and phonetic encoding of complex articulatory movements for both speaking and signing. These same regions were also reliably activated by the complex oral and limb motor tasks, suggesting that language formulation was not required to engage these anterior brain regions. Of course, this finding does not imply that these anterior cortical regions only play a motor-articulatory role in language production—rather, they point to their multifunctionality, particularly the frontal operculum (cf. Grodzinsky and Amunts, 2005). In contrast, bilateral posterior brain regions (posterior superior and middle temporal gyri, posterior superior temporal sulcus, and angular gyrus) were only engaged during language production and not during complex motor baseline tasks. Braun et al. (2001) suggest that these bilateral posterior brain regions are involved in semantic and pragmatic processes required to create autobiographical narratives in both ASL and English.

Emmorey et al. (2007) conducted a conjunction analysis for single sign production (by native deaf ASL signers) and single word production (by hearing English speakers) in a picture-naming task, with a baseline task that required participants to make an orientation judgment (upright or inverted) for unknown faces, overtly signing or saying *yes* or *no* on each trial. Consistent with the Braun et al. (2001) results, both sign and speech engaged the left inferior frontal gyrus (Broca's area) indicating a modality-independent role for this region in lexical production. Using probabilistic cytoarchitectonic mapping and data from the Braun et al. (2001) study, Horwitz et al. (2003) reported that BA 45 was engaged during both speaking and signing, but there was no involvement of BA 44, compared to the motor baseline conditions. In addition, there was extensive activation in BA 44, but not in BA 45, for the non-linguistic oral and manual control tasks compared to rest. This pattern of results suggests that BA 44, rather than BA 45, is engaged during the production of complex movements of the oral and manual articulators and that BA 45 is more likely engaged in articulator-independent aspects of language production. Finally, Emmorey et al. (2007) found that both sign and word production engaged left inferior temporal regions, which have been shown to be involved in conceptually driven lexical access (e.g., Indefrey and Levelt, 2004).

Overall, these conjunction studies, along with additional data from lesion and neuroimaging studies, indicate that sign and speech production both rely on a primarily left lateralized neural network that includes left inferior frontal cortex (BA 44/45, 46, and 47), pre-SMA, insula, middle/inferior temporal cortex, and inferior parietal cortex (see also Hickok et al., 1996; Corina et al., 2003; Kassubek et al., 2004). We point out that our null findings for the direct contrast between speech and signing in this left lateralized network are consistent with the conjunction study results.

## Experiment 2: contrasting the neural substrate for signed vs. spoken language comprehension

As with language production, few studies have directly contrasted signed and spoken language comprehension. An early PET study by Söderfelt et al. (1997) presented hearing bilinguals with short, signed narratives (Swedish Sign Language) and audiovisually presented spoken Swedish narratives (a video of the same model speaking). The direct contrast revealed greater activation in bilateral perisylvian cortex for audiovisual speech comprehension and greater activation in bilateral middle/inferior temporal cortex (BA 37, 19) for sign language comprehension, reflecting auditory neural responses for speech perception and visual motion processing for sign perception. No other differences were reported, but this study was underpowered with only six participants and without the spatial resolution and sensitivity of modern fMRI. For example, it is possible that parietal cortex may have been more involved in signed than spoken language comprehension given the role of parietal cortex in sign production and in the recognition of human actions (e.g., Corina and Knapp, 2006), but the Söderfelt et al. (1997) study may have been unable to detect this difference. Neuroimaging studies that have separately examined sign language comprehension (by deaf or hearing signers) and audiovisual spoken language comprehension have observed more parietal activation for sign comprehension (e.g., MacSweeney et al., 2002a). Here we report the first direct contrast (to our knowledge) between the comprehension of sign language and audiovisual spoken language by hearing native ASL-English bilinguals. We also report the first conjunction analysis (to our knowledge) that identifies the neural overlap between the two languages for these bilinguals.

### Materials and methods

#### Participants

Thirteen hearing native ASL-English bilinguals (9 females; mean age = 26.4 years; *SD* = 4.7 years) participated in the study. All participants were born into deaf signing families, were right handed, and had normal or corrected-to-normal vision by self-report. ASL data from these participants was presented in McCullough et al. (2012).

#### Materials and procedure

The spoken language materials are from Saygin et al. (2010) and consisted of audiovisual English sentences produced by a female native speaker that expressed motion (e.g., “The deer jumped over the brook”), static location (e.g., “Her family lives close to the river”), or metaphorical (fictive) motion (e.g., “The hiking trail crossed the barren field”). Co-speech gestures were not produced. The signed language materials are from McCullough et al. (2012) and consisted of similar (but non-identical) ASL sentences produced by a male native signer that expressed motion (e.g., English translation: “Many dogs were running loose around the farmyard”) or static location (e.g., English translation: “The lion slept in his enclosure at the zoo”). For the purposes of this study, sentence type was not a treated as a variable of interest.

Presentation of English and ASL sentences was counter-balanced across participants. Participants pressed a button when they heard/saw a sentence that was semantically anomalous (e.g., “The wooden fence crosses the late curfew.”). Anomalous sentences were relatively rare, occurring either once or never within a block, and frequency was matched across languages (12% of sentences were anomalous for both ASL and English). The baseline condition for ASL consisted of video clips of the model signer sitting in the same position but not signing, and participants decided whether the color of a black dot superimposed on the model's chin changed to white during the baseline. The baseline condition for English was parallel: participants saw video clips of the same speaker sitting in the same position, but remaining silent and with a dot superimposed on her chin. Participants monitored whether a continuous pure tone presented along with the video stimuli changed frequency, and the change in frequency occurred simultaneously with the change in dot color. The (in)frequency of the dot targets was matched with the sentence condition targets (12%). These low-level baseline conditions presented visual (and auditory) stimuli along with a simple button press task to provide a reference against which to measure neural responses to the English and ASL sentences.

#### MRI data acquisition and analysis

MRI data were collected using a 3-Tesla GE Signa Excite scanner equipped with an eight-element phased-array head coil at the Center for fMRI at the University of California, San Diego. For each participant, a 1 × 1 × 1.3 mm anatomical scan was collected, usually in the middle of the scanning session. Echo-planar volumes were acquired from the whole brain with a repetition time (TR) of 2000 ms, an echo time (TE) of 30 ms, 3.5 mm in-plane resolution, and 4 mm slice thickness (no gap). Image preprocessing and statistical analyses were performed using Analysis of Functional Neuroimages (AFNI) software package (version AFNI_2010_10_19_1028; Cox, 1996). Further details on data acquisition and pre-processing can found in Saygin et al. (2010) and McCullough et al. (2012).

For the individual-level analysis, ASL and English sentence blocks were modeled as regressors of interest in the design matrix with respect to the control baseline. The design matrix was constructed using AFNI's 3dDeconvolve. Six motion parameters, obtained during head motion correction (AFNI's 3dvolreg), and a Legendre polynomial set ranging from zero to third order to account for slow drifts were included in the design matrix as nuisance regressors. The regressor of interest beta values and *t*-values from each individual were estimated and calculated using AFNI's 3dREMLFIT (Chen et al., 2012). For the group-level analysis, individuals' voxelwise betas and their corresponding *t*-values for each contrast of interest served as inputs to group-level, mixed-effects meta-analysis (AFNI's 3DMEMA, Chen et al., 2012). We used false discovery rate correction for multiple comparisons to identify clusters of significant activation in the ASL vs. English sentence contrast. Only clusters of 30 or more contiguous voxels surviving *q* = 0.001 are reported.

To identify the regions of the common activation between ASL and English sentence comprehension relative to the baseline, a conjunction analysis was performed using the minimum statistic (*q* = 0.01) for each condition to test the conjunction null hypothesis (i.e., minimum statistic compared to conjunction null; Nichols et al., 2005).

### Results

**Figure 2:**
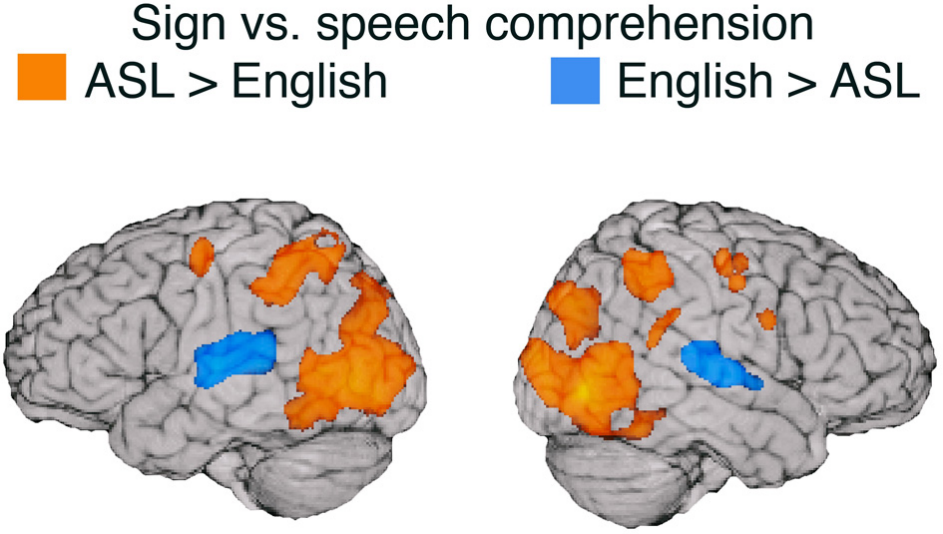
**Illustration of the contrast (thresholded at *q* = 0.001) between signed and spoken language comprehension overlaid on an individual human brain**. Regions more active for spoken language comprehension (indicated in blue) include superior temporal gyrus in both hemispheres. Regions more active for signed language comprehension (in orange) include bilateral middle occipital cortex, bilateral pre-central gyrus, bilateral post central gyrus, bilateral IPS, left SPL, left SMG, and right pSTG. Up to 30 mm beneath the surface of cortex is displayed on the contrast map.

Table 2 lists the peak Talairach coordinates and cluster volumes for the contrast between ASL and English, and the results are illustrated in Figure 2. Only the STG (bilaterally) was more active for comprehension of spoken than signed language. In contrast, several regions were more active for the comprehension of signed than spoken language: bilateral posterior middle temporal cortex (extending into lateral occipital cortex), bilateral inferior and superior parietal cortices (more extensive on the left), and bilateral premotor cortex.

**Table 2:** **Peak Talairach coordinates and cluster volumes for the contrast between sign and spoken language comprehension (*q* = 0.001)**.

**Region**	**Side**	***X***	***Y***	***Z***	**Vol mm^3^**	***T***
**SIGN > SPOKEN LANGUAGE**						
**Frontal cortex**						
Pre-central gyrus (BA 4)	L	−23	−11	+50	964	6.09
	R	+50	+2	+26	281	6.14
	R	+34	−12	+46	972	5.93
**Temporal cortex**						
Posterior superior temporal gyrus (BA 22)	R	+57	−40	+23	589	6.26
**Parietal cortex**						
Post central sulcus (BA 3)	L	−27	−46	+44	4161	6.96
	R	+30	−46	+44	3648	7.83
Intraparietal sulcus (BA 39)	L	−24	−76	+29	2221	6.75
	R	+28	−76	+30	2804	8.53
Superior parietal lobule (BA 7)	L	−19	−61	+48	1188	6.73
Supramarginal gyrus (BA 40)	L	−53	−35	+37	527	5.77
**Occipital cortex**						
Middle occipital gyrus (BA 19)	L	−36	−70	+4	17172	13.09
	R	+41	−69	+1	17120	24.76
**SPOKEN > SIGN LANGUAGE**						
**Temporal cortex**						
Superior temporal gyrus (BA 22)	L	−42	−23	+9	7603	−10.62
	R	+50	−20	+8	5647	−8.55

### Discussion

Replicating Söderfelt et al. (1997), the audio-visual signal for speech activated the STG bilaterally to a greater extent than the purely visual signal for sign language for hearing ASL-English bilinguals (see Figure 2). Although there is evidence that visual stimuli (including sign language) activate auditory cortex for deaf people (e.g., Finney et al., 2001; Cardin et al., 2013), comprehending spoken language for hearing individuals requires significantly more neural resources and sustained activation in auditory cortices compared to comprehending sign language (see also Leonard et al., 2012). In addition, MacSweeney et al. (2002a) found that hearing native users of British Sign Language (BSL) exhibited less extensive activation along left STG compared to deaf native signers when comprehending BSL sentences and hypothesized that auditory processing of speech has privileged access to more anterior regions of STG (adjacent to primary auditory cortex), such that hearing signers engage this region much less strongly during sign language processing (see also Emmorey and McCullough, 2009).

Not surprisingly, ASL comprehension engaged bilateral occipito-temporal cortex to a much greater extent than comprehension of audio-visual English. Activation in posterior middle temporal cortex (including area MT+) likely reflects perception of the much larger movements of the hands and arms produced within a larger physical space for sign language, compared to the perception of the relatively small mouth movements of speech. Sign language movements also have a larger spatial frequency and thus are more likely to involve extra-foveal visual processes. Our findings replicate the results of other between-group studies that compared sign language comprehension by signers and audiovisual speech comprehension by hearing monolingual speakers, using relatively low-level baselines (e.g., MacSweeney et al., 2002a; Courtin et al., 2011).

Of particular interest is the extensive activation in bilateral parietal cortices observed for sign language comprehension relative to spoken language (see Figure 2). One partial explanation for greater parietal activation for ASL may lie in the semantic content of the sentences presented in the study—the sentences in both ASL and English conveyed information about the movement or static location of a referent. The ASL sentences involved classifier constructions in which locations in signing space correspond to referent locations and movements of the hand(s) through space depict the movements of a referent. Previous research has found that understanding this type of spatial language recruits left parietal cortex (the intraparietal sulcus) to a greater extent for ASL or BSL than for spoken English (MacSweeney et al., 2002b; McCullough et al., 2012). In addition, right parietal damage can impair comprehension of these types of spatial expressions (but does not cause sign aphasia), suggesting a critical role for the right hemisphere in comprehending spatial language in which physical space is used to express spatial concepts (e.g., Emmorey et al., 1995; Atkinson et al., 2005). In addition, the production of location and motion expressions using classifier constructions differentially recruits bilateral superior parietal cortex compared to the production of lexical signs (nouns) and compared to the production of lexical prepositions in spoken English (Emmorey et al., 2005, 2013).

Parietal cortex may also play a distinct role in phonological processing and working memory for sign language. Direct stimulation of the left supramarginal gyrus (SMG) results in handshape substitutions during picture naming (Corina et al., 1999), and MacSweeney et al. (2008b) reported greater activation in left SMG (extending into the superior parietal lobule) when deaf signers made phonological judgments about signs (do they share the same location?) than when they made phonological (rhyming) judgments about words, despite SMG engagement by both tasks relative to a baseline. Working memory for sign language also appears to engage parietal regions to a greater extent than for spoken language (Rönnberg et al., 2004; Buchsbaum et al., 2005; Bavelier et al., 2008; Pa et al., 2008). In particular, storage (the phonological buffer) and maintenance (rehearsal) of signs appear to rely more on parietal cortex compared to storage and maintenance processes for words.

Furthermore, the bilateral premotor and inferior parietal regions that were more active for sign than speech comprehension in Experiment 2 correspond to the predictive component of the Action Observation Network (AON), which is engaged when observing non-linguistic human body actions (e.g., Buccino et al., 2001; Caspers et al., 2010). The proposed function of the premotor-parietal (dorsal) component of the AON is the generation of predictions for observed manual actions (Kilner, 2011). Predictive coding accounts of the AON propose that premotor and parietal cortices (the motor system used to produce manual actions) is active during action observation because it generates internal models that can be used to predict incoming visual input (Kilner, 2011; Schippers and Keysers, 2011). Premotor and parietal cortices are more engaged during active action understanding than during passive viewing of actions (Schippers and Keysers, 2011). Similarly, although several studies report prefrontal and parietal activation during active comprehension of signed sentences (e.g., Neville et al., 1998; MacSweeney et al., 2002a; Sakai et al., 2005) and single signs (e.g., MacSweeney et al., 2006), Emmorey et al. (2011) found little activation in these regions when deaf signers passively viewed strings of ASL signs. For sign language, this premotor-parietal circuit may be engaged in predicting the incoming visual input as part of active language comprehension.

Such a hypothesis is consistent with recent work by Pickering and Garrod (2013) who view language production and comprehension as forms of action and action perception, respectively. Applying forward modeling frameworks developed for human action to language comprehension, they propose that comprehenders use covert imitation and forward modeling to predict upcoming utterances. In this model, production and comprehension are integrated systems and both involve the extensive use of prediction. Perceivers of language construct forward models of others' linguistic actions that are based on their own potential actions. Thus, the differential premotor and parietal activation observed for sign language comprehension may be tied to the distinct neural substrate that supports sign language production.

### Common neural substrates for signed vs. spoken language comprehension

**Figure 3:**
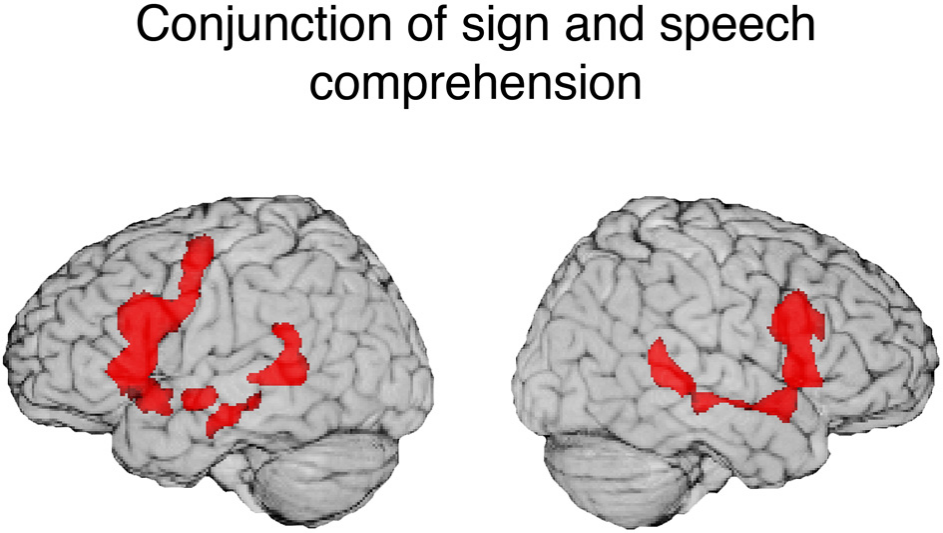
**Illustration of the conjunction for signed and spoken language comprehension**. The red regions (overlaid onto an individual human brain) were active for both signed and spoken language comprehension relative to the baseline (threshold at *q* = 0.01). Conjunction of common activations up to 30 mm beneath the surface of cortex are displayed.

To identify shared neural substrates for sign and speech comprehension, we conducted a conjunction analysis for the contrast between each language and its baseline. The results are listed in Table 3 and illustrated in Figure 3. Comprehension of both ASL and English engaged a bilateral fronto-temporal neural network, encompassing the inferior frontal gyrus (extending along the pre-central gyrus in the left hemisphere) and the superior temporal sulcus (extending into posterior STG in the left hemisphere).

**Table 3:** **Center of mass Talairach coordinates and cluster volumes for the conjunction of sign and spoken language comprehension (each vs. its baseline; thresholded at *q* = 0.001)**.

**Region**	**Side**	***X***	***Y***	***Z***	**Vol mm^3^**
**FRONTAL CORTEX**					
Inferior frontal gyrus (BA 45, 44, 4)	L	−38	+11	+19	13145
(BA 45, 44)	R	+42	+13	+17	7876
Medial frontal gyrus (BA 6)	L/R	+1	+3	+53	3286
**TEMPORAL CORTEX**					
Superior temporal sulcus (BA 22, 21)	L	−47	−8	−7	751
	L	−48	−41	+8	3486
	R	+50	−26	+1	4080
Hippocampus	L	−34	−19	−13	899
Parahippocampal gyrus (BA 36)	L	−19	−28	−14	99

One striking result from the conjunction analysis is the degree to which modality independent activation during language comprehension is bilateral. Although reading print is highly left-lateralized, auditory and audio-visual spoken language comprehension engages a more bilateral network (e.g., Price, 2012). MacSweeney et al. (2002a) reported very similar bilateral fronto-temporal activation for comprehension of BSL sentences by native signers and comprehension of audiovisual English sentences by hearing native speakers. In contrast, Neville et al. (1998) observed left lateralized activation for hearing speakers reading English sentences, but bilateral activation for native signers comprehending ASL sentences. These findings highlight the importance of comparing sign language comprehension which always involves face-to-face interaction with the comprehension of audio-visual speech rather than with reading text or with a disembodied auditory-only speech signal (see also Hickok et al., 1998).

According to the dual stream model of speech processing proposed by Hickok and Poeppel (2007), phonological-level processing and representation of speech is associated with middle to posterior portions of bilateral STS, with asymmetric functions in the left and right hemispheres. They suggest that left STS is more engaged in temporal and categorical processing of segment-level information, while right STS is more engaged in processing suprasegmental, prosodic information. Evidence that posterior STS might also be engaged in phonological processing for sign language comes from studies that examined linguistically structured pseudosigns. Pseudosigns have phonological structure for signers but do not access semantic or syntactic representations. A PET study by Petitto et al. (2000) found that viewing pseudosigns (as well as real signs) engaged STS bilaterally for deaf signers, but no activation was observed for hearing individuals who had not acquired a sign-based phonological system. Similarly, an fMRI study by Emmorey et al. (2011) reported that pseudosigns activated left posterior STS to a greater extent for deaf ASL signers than for hearing non-signers. Increased left posterior STS activation for signers is hypothesized to reflect heightened sensitivity to temporal body movements that conform to the phonological structure of ASL since dynamic movements (e.g., path movements or changes in hand orientation) are critical to identifying syllabic structure in sign languages. Left STS may be significantly more active for signers than for non-signers because neurons in this region become particularly receptive to segment-level body movements that are linguistically structured and constrained. Right STS may also be engaged in sign-based phonological processing but perhaps only at the sentential level. An intriguing possibility is that left STS subserves categorical and combinatorial processing of sublexical sign structure, while right STS subserves more global phonological processes (e.g., sentential prosody expressed by movement; see Newman et al., 2010).

In addition, for spoken language, bilateral STS interfaces with MTG by mapping phonological representations onto lexical conceptual representations (the dorsal stream in the Hickok and Poeppel model). A similar interface may occur for signed language. The conjunction analysis revealed that STS activation extends into middle MTG for both ASL and English comprehension (see Figure 3). Results from a recent MEG study by Leonard et al. (2012) indicate that STS is engaged for both ASL signs and English words (in a sign/word picture matching task) during a relatively late time window associated with lexical-semantic processing (300–500 ms after stimulus onset), but only speech for hearing individuals activated STS during early sensory processing (80–120 ms). This finding suggests that STS activation for sign language is associated with lexical retrieval processes, rather than with early sensory processing which may be modality specific. Thus, bilateral activation in STS (extending into MTG) observed for both sign and speech may reflect amodal lexical-semantic and sublexical (phonological-level) processes (see Berent et al., 2013, for evidence for amodal phonological processes across signed and spoken languages).

Consistent with previous between-group studies, comprehension of audiovisual sentences and signed sentences both activate bilateral inferior frontal cortex, with activation extending anteriorly and dorsally in the left hemisphere. Comprehension functions associated with left inferior frontal cortex are numerous and are likely shared by both signed and spoken languages, e.g., syntactic processing, semantic retrieval, phonological-lexical integration—unification processes in Hagoort's (2013) model of language processing. Shared comprehension functions that may be associated with right inferior frontal cortex include prosodic processing and semantic inferencing (likely involved in the semantic anomaly detection task used here).

In sum, the conjunction results indicate that sign and audio-visual speech comprehension rely on a bilateral fronto-temporal network, with a slight left-hemisphere bias. The superior temporal sulcus is likely engaged in modality-independent phonological and lexical-semantic processes. Left inferior and middle frontal cortex may be engaged in various aspects of amodal syntactic, phonological, and semantic integration, while the right hemisphere homologue of Broca's area (BA 44/45) may be involved in semantic interpretation and sentence-level prosodic processing for both sign and speech comprehension.

## Summary and conclusions

The direct contrasts between ASL and English for both production and comprehension revealed relatively large differences in neural resources related to perceptual and motor features of these two languages for hearing bimodal bilinguals (see Figures 1, 2). In contrast, direct contrasts between two spoken languages for unimodal bilinguals do not reveal such dramatic differences in neural activation (Gullberg and Indefrey, 2006). We suggest that the surface level differences between signed and spoken languages should not be dismissed as uninteresting and that these differences are critical for understanding how sensory-motor systems impact psycholinguistic processes and the underlying neural substrate for language.

A key psycholinguistic difference between signed and spoken language production is the role of perceptual (auditory or visual) feedback in monitoring language output and in learning new articulations for both adults and children (Emmorey et al., 2009a,c). Speakers use auditory feedback to detect errors (Postma and Noordanus, 1996) and to compare to “auditory targets” in the acquisition and maintenance of phonetic aspects of spoken segments and syllables (e.g., Guenther et al., 1998). Neural responses reflecting auditory feedback were observed in bilateral STC, which was significantly more engaged during speech than sign production as participants heard their own voices. In addition, we hypothesize that greater activation in right frontal cortex may reflect error detection processes involved in auditory monitoring of speech output as proposed within the DIVA modal of speech production (Tourville and Guenther, 2011).

For sign production, visual feedback cannot be easily parsed by the comprehension system (self-produced signs are not recognized very accurately; Emmorey et al., 2009a). In addition, “visual targets” are problematic for sign production because visual input from one's own signing differs substantially from visual input from another's signing. Thus, it is likely that signers rely on somatosensory more than visual feedback to monitor for errors and to acquire and maintain sign productions. Greater activation along the post-central gyri and anterior superior parietal cortex for signing compared to speaking may reflect somatosensory feedback received from signing.

The unexpected finding of increased activation in bilateral occipital cortex for speaking compared to signing may also be related to differences in sensory feedback. Specifically, we hypothesize that greater occipital activation for speaking may actually be due to suppression of cortical activity during signing. We speculate that cortical attenuation in visual cortex may serve to distinguish between visual stimulation arising from the signer's own movements and externally produced movements toward the body and face. Predicting the visual consequences of one's own actions may attenuate activation in visual cortex, which would help to dissociate sensory signals generated by one's own actions from sensory signals that are externally generated by the environment.

An important finding from these studies is that *both* sign language production and comprehension engaged parietal cortex to a greater extent than spoken language. In fact, the peak coordinates within the anterior superior parietal lobule (in the post-central sulcus) are within 10 mm of each other for sign production (−35, −31, +50; +33, −35, +51) and sign comprehension (−27, −46, +44; +30, −46, +44). We hypothesize that anterior SPL (possibly in conjunction with inferior parietal cortex) is more engaged during sign language comprehension because the *production* system for signing differs from speech and that production and comprehension are interweaved for sign language, as has been proposed for spoken language (Pickering and Garrod, 2013). Specifically, parietal regions may be involved in creating a forward model that predicts the incoming visual manual signal during comprehension. Recently, Hosemann et al. (2013) provided ERP evidence suggesting that sentence comprehension in sign language (in this case, German Sign Language) involves the use of forward modeling such that manual information in a transitional movement is used to predict an upcoming sign. Consistent with these results, the MEG study by Leonard et al. (2012) reported a larger response in left parietal cortex (in the intraparietal sulcus) for incongruent signs (those that did not match a preceding picture) than congruent signs, but no difference in parietal cortex was observed for spoken language. Thus, evidence is mounting that parietal cortex may be involved in internal simulations (generating predictions) during sign language comprehension. Internal simulations differ between spoken and sign languages because their production systems involve different articulators.

The conjunction results point to neural substrates that support modality-independent, shared computational processes for spoken and signed languages. The conjunction studies for production that were reviewed here along with the results from the comprehension conjunction (Figure 3) identify left inferior frontal cortex as a key amodal language area. For production, the left pre-central gyrus and the supplementary motor area have been found to be jointly engaged when signing or speaking (and when covertly signing or speaking in a rehearsal task—see Pa et al., 2008), pointing to an amodal role in the complex articulations required by the human language system. For comprehension, bilateral STS was engaged for both English and ASL, and we hypothesize there may be similar asymmetric functions for left and right STS for both language types. Left anterior STS regions may be engaged in amodal syntactic processes (e.g., Friederici et al., 2003), while posterior STS regions may be involved in lexical-phonological processes that are independent of modality. Right STS may function to integrate suprasegmental, prosodic information conveyed either by vocal intonation or intonation expressed by facial expressions and manual prosody (see Sandler, 1999; Dachovsky and Sandler, 2009, for evidence of visual prosody in sign languages). Right inferior frontal cortex was also engaged during spoken and sign language comprehension and may be involved in semantic processing, as well as prosodic segmentation during sentence comprehension (cf. Friederici, 2011).

In sum, results from direct contrasts between signing and speaking and between visual and audio-visual language comprehension revealed non-obvious distinctions between the two language types. The differences between sign and speech were not restricted to input/output differences in primary sensory and motor systems—surface level differences were also observed in heteromodal association cortex, suggesting that higher order systems may be needed to integrate modality-specific information. Our conjunction analysis revealed the expected overlap in left perisylvian language regions but also indicated an important role for the right hemisphere during face-to-face language comprehension. Further detailed studies that target specific linguistic processes are needed to identify invariant structure-function associations within the language network and to demarcate the specific functional roles of cortical regions that distinguish between languages by hand and languages by mouth.

### Conflict of interest statement

The authors declare that the research was conducted in the absence of any commercial or financial relationships that could be construed as a potential conflict of interest.

## Footnotes

^1^The frequency manipulation generated only weak differences in neural activity, and the results reported here are collapsed across frequency. Word length and sign length were also manipulated (one syllable vs. two syllables), but no significant length effects were observed for either language, and the reported results are collapsed across word/sign length.
