# Supplementary material for: How sensory-motor systems impact the neural organization for language: direct contrasts between spoken and signed language
Source: Front Psychol. 2014 May 27;5:484. doi: 10.3389/fpsyg.2014.00484 (PMC4033845; doi:10.3389/fpsyg.2014.00484)
Supplement: Supplementary file 1 [file DataSheet1.DOCX]

Supplementary Table. Summary of conjunction results using data from Emmorey et al. (2005). Activation peaks reflect *t*-values from subjects in the Emmorey et al. (2005) study; critical *t*(67) = ±4.9.

| Region | Side | X | Y | Z | *t* |
| --- | --- | --- | --- | --- | --- |
| **Signing > Speaking** |  |  |  |  |  |
| Frontal Cortex |  |  |  |  |  |
| Medial frontal gyrus (BA 6) | R | +12 | –9 | +54 | +10.19 |
| Precentral gyrus (BA 6) | R | +34 | –19 | +59 | +13.92 |
| Temporal Cortex |  |  |  |  |  |
| Middle temporal gyrus (BA 37) | L | –45 | –65 | +9 | +8.43 |
| Parietal Cortex |  |  |  |  |  |
| Inferior parietal cortex (BA 40) | L | –31 | –38 | +56 | +26.23 |
|  | R | +35 | –37 | +50 | +16.82 |
|  | R | +51 | –31 | +39 | +10.16 |
| Occipital Cortex |  |  |  |  |  |
| Cuneus (BA 19) | L | –8 | –83 | +34 | +8.76 |
| Cerebellum |  |  |  |  |  |
|  | L | –24 | –43 | –22 | +11.52 |
|  | R | +32 | –41 | –28 | +17.85 |
|  | R | +21 | –45 | –20 | +19.75 |
|  |  |  |  |  |  |
| **Speaking > Signing** |  |  |  |  |  |
| Frontal Cortex |  |  |  |  |  |
| Superior frontal gyrus (BA 10) | R | +10 | +58 | –3 | +5.79 |
| Superior frontal gyrus (BA 9) | L | –10 | +55 | +36 | +6.54 |
|  | L | –24 | +51 | +33 | +5.88 |
| Medial frontal gyrus (BA 11) | R | +10 | +26 | ­–9 | +7.47 |
| Temporal Cortex |  |  |  |  |  |
| Superior temporal gyrus (BA 41) | L | –56 | –19 | +3 | +20.16 |
|  | R | +57 | –22 | +5 | +19.37 |
| Occipital Cortex |  |  |  |  |  |
| Cuneus (BA 18) | R | +25 | –91 | –2 | +6.01 |
| Inferior occipital gyrus (BA 17) | L | –22 | –92 | –12 | +7.51 |
